# Facile Controlling of the Physical Properties of Zinc Oxide and Its Application to Enhanced Photocatalysis

**DOI:** 10.1155/2021/5533734

**Published:** 2021-04-10

**Authors:** Tuan Vu Anh, Thi Anh Tuyet Pham, Van Hung Mac, Thanh Hung Nguyen

**Affiliations:** School of Chemical Engineering, Hanoi University of Science and Technology, Hanoi, Vietnam

## Abstract

In this study, the physical properties of ZnO were facile controlled by the synthesis method with the addition of capping and precipitation agents. As-prepared ZnO samples had different morphologies such as carnation flower-like ZnO (CF-ZnO), rose-flower-like ZnO (RF-ZnO), rod-like ZnO (R-ZnO), and nanoparticle ZnO (N-ZnO) and were characterized by SEM, XRD, N_2_ adsorption/desorption isotherms, FT-IR, and DR/UV-vis. All samples had a crystallite structure of hexagonal wurtzite type. The CF-ZnO and RF-ZnO samples had the hierarchical structure like a carnation flower and a beautiful rose, respectively. R-ZnO was composed of many hexagonal rods and few spherical particles, while N-ZnO microstructures were made up of nanoparticles with approximately 20–30 nm, exhibiting the largest surface area, pore volume, and pore width among as-prepared samples, and their crystal size and bandgap energy were 17.8 nm and 3.207 eV, respectively. The catalytic performances of ZnO samples were evaluated by degradation of Tartrazine (TA) and Caffeine (CAF) under low UV irradiation (15 W). N-ZnO showed a high photocatalytic activity compared to other samples. Besides, the reaction kinetics was investigated by the first-order kinetic model, and the catalytic performance of ZnO was evaluated through several organic pollutants.

## 1. Introduction

The rapid development of industry has resulted in the release of a large amount of toxic gas and wastewater into the environment. Particularly, in developing countries, wastewater from factories and residents is a remarkable problem because it is harmful to human health and the ecosystem if it is directly discharged into the environment [1]. From the UN World Water Development Report, in many developing countries, more than 70 percent of unprocessed industrial waste is discharged into water sources and contaminates water [2]. The largest sources of hazardous industrial wastewater come from mining, pulp mill, textiles, rubber, tanning, sugar mills, and pharmaceutical manufacture [3]. Therefore, industries need to apply a suitable method for treating wastewater for that it can be accepted within the permissible standards. To either degradation or adsorption of persistent organic compounds from effluents, ZnO is one of the candidate materials used in practical applications [4].

Zinc oxide (ZnO) has been of great interest because of its excellent physical properties, such as low cost, nontoxicity, high thermal stability, photosensitivity, advanced optical properties, and environmentally friendly features [5, 6]. Therefore, ZnO has been widely used in many fields, such as adsorption, paint, cosmetic, superconductor, and catalyst [7–12]. As an effective photocatalyst in the degradation of persistent organic compounds [13–15] and catalyst support [16, 17], ZnO should have a sufficient structure area and morphology to allow the diffusion of active species and electron transfer [18]. Well-designed ZnO has shown high performance in the degradation of dyes as promised photocatalyst [19, 20].

ZnO with the wide bandgap energy (3.37 eV) and the high exciton binding energy (60 meV) could absorb a larger fraction of the UV spectrum to oxidize harmful organic substances in wastewater effectively [21–23]. The photocatalytic mechanism for degradation of organic pollutants of ZnO nanomaterials has been indicated in previous studies [12, 24–26]. Upon the UV irradiation with the photonic energy (*hʋ*) equal to or greater than the bandgap energy (*E*_*g*_) of ZnO, the electrons in the valence band can be excited (*e*−) and transfer to the conduct band, leading to holes (*h*^+^) generation in the VB. The electrons can active molecular oxygen to form superoxide anion radicals ^•^O_2_^−^, whereas the photogenerated holes react with either water (H_2_O) or hydroxyl ions (OH^−^) to produce hydroxyl radicals (^•^OH). The formation of ^•^OH, ^•^O_2_^−^ radicals, which are powerful oxidizing agents, will attack the pollutants adsorbed on the surface of ZnO through hydroxylation, oxidation, and mineralization processes to create finally harmless compounds of CO_2_ and H_2_O. However, electrons and holes can recombine with the release of heat or photons, leading to the reduction of quantum yield for the photocatalytic degradation of organic pollutants process. The recombination rate is strongly influenced by various parameters with correlation to the nature of the ZnO nanostructure [27].

It was reported that the photocatalytic activity of ZnO was strongly dependent on the morphology and crystallinity, which is highly dependent on the synthesis method. The appropriate shape, morphology, and dimensionality will give an advantage for the transfer and separation of photogenerated electrons and holes, promoting the in situ generation of reactive transitory species as well as facilitating the adsorption and diffusion on the surface of ZnO, resulting in efficient photodegradation of organic compounds [26, 28–32]. Nanosize ZnO synthesized by the facile and fast method showed the highly effective degradation of tartrazine under UV light [26]. The hierarchical flower-like ZnO synthesized by the simple hydrothermal process could degrade caffeine under UV-light irradiation, and the degradation efficiency for caffeine solution of 5 mg/L was up to 97.6% within 120 min [12]. Nanowire ZnO prepared by the coprecipitation at the low temperature was applied to acid red 57, and the degradation efficiency was 90.03% within 200 min at the acid red 57 concentration of 30 mg/L [33].

There are no detailed studies on the synthesis and comparison of photocatalytic performance ZnO with different physical properties and morphologies. In this study, ZnO samples with the different morphologies were prepared by the simple precipitation and hydrothermal method by using the capping agents trisodium citrate, urea, and hexamethylene tetramine. As-prepared ZnO samples were characterized by FE-SEM, XRD, BET, FT-IR, and DR/UV-Vis. The effect of physical properties on the catalytic efficiency of ZnO was evaluated by degradation of TA and CAF in a batch reactor under UV-light irradiation. This study can clearly show the effect of the morphologies and structures of ZnO nanoparticles on their photocatalytic efficiency. In addition, the reaction kinetic was investigated by the first-order kinetic model, and the catalyst performance of ZnO was evaluated by several organic pollutants.

## 2. Experimental

### 2.1. Materials

Tartrazine (TA, 99%) and caffeine (CAF, 99%) were purchased from Sigma-Aldrich, and the physical properties of TA and CAF are presented in Table 1. Zn(NO_3_)_2_.6H_2_O (99%), urea ((NH_2_)_2_CO, 99.5%), hexamethylene tetramine (HMTA, C_6_H_12_N_4_, 99%), and sodium citrate (C_6_H_5_NaO_7_.2H_2_O, 99%) were purchased from Merk. All the chemicals were used without any purification, and distilled water was used throughout all experiments.

### 2.2. Preparation of Samples

#### 2.2.1. Preparation of CF-ZnO

6 mmol of Zn(NO_3_)_2_.6H_2_O and 0.6 mmol of trisodium citrate were dissolved in 85 mL of distilled water and stirred in 10 min. Then, 15 mL of NaOH 2M was directly added to the solution. After 120 min stirring, the white precipitate was filtered and washed several times with distilled water and ethanol to remove the excess sodium citrate and bases. The white powder was dried at 80°C for 24 h and calcined at 400°C for 2 h at the heating rate of 2°C/min to obtain carnation-flower-like ZnO (CF-ZnO).

#### 2.2.2. Preparation of RF-ZnO

1.5 mmol of Zn(NO_3_)_2_.6H_2_O and 0.03 mol of urea were dissolved in 100 mL of distilled water. After stirring for about 30 min, the solution was poured into a 120 mL Teflon-lined stainless-steel autoclave and then heated to 90°C for 24 h. The white precipitate was washed with distilled water several times and dried at 80°C for 24 h. Finally, the rose-flower-like ZnO (RF-ZnO) was obtained by calcining the precipitate at 400°C for 2 h at the heating rate of 2°C/min.

#### 2.2.3. Preparation of R-ZnO

0.1 mol of Zn(NO_3_)_2_.6H_2_O and 0.1 mol of HMTA were dissolved in a beaker with 100 mL of distilled water and stirred in 20 min. The solution was heated up to 90°C and kept at this condition for 1 h. The white precipitate was filtered and washed several times by distilled water and then dried at 80°C for 24 h. Finally, the rose-flower-like (R-ZnO) was obtained by calcining at 400°C for 2 h at the heating rate of 1°C/min.

#### 2.2.4. Preparation of N-ZnO

Nano-ZnO was modified from the synthesis method of R-ZnO with the addition of sodium citrate. 0.1 mol of Zn(NO_3_)_2_.6H_2_O, 0.1 mol of hexamethylene tetramine and 0.01 mol of sodium citrate were dissolved in a beaker with 100 mL distilled water and stirred in 20 min to form the clear solution. Then, a similar procedure of R-ZnO was applied to obtain N-ZnO.

### 2.3. Characterization

The crystal phase of samples was investigated by X-ray powder diffraction. XRD patterns were obtained by using a Bruker D8 Advance diffractometer (Germany) with the Cu K_*α*_ irradiation (40 kV, 40 mA). The 2*θ* ranging from 10 to 80° was selected to analyze the crystal structure. The morphology of the samples was observed by the emission scanning electron microscopy (FE-SEM, JEOL-7600F). The textural properties were measured via N_2_ adsorption/desorption isotherms using a micromeritics (Gemini VII). The specific surface area was obtained by using the Brunauer–Emmett–Teller (BET) method, and the pore volume and pore diameter were determined by the Barrett, Joyner, and Halenda (BJH) method. UV-vis diffuse reflectance spectra (DR/UV-vis) of the as-synthesized samples were measured on a UV-Vis spectrometer (Avantes).

### 2.4. Photocatalytic Activity Test

The experimental degradation of dye was conducted in a batch reactor under low UV irradiation (15 W). Typically, a certain amount of catalyst was added to a beaker with 100 mL of dye solution at desired pH under magnetic stirring. The pH was adjusted by 0.1 M HCl and 0.1 M NaOH solutions. At a given time interval, an aliquot (2 mL) of dye was withdrawn from the suspension and immediately filtered through a Millipore filter (0.45 *μ*m PTFE membrane) to separate solid particles. The dye concentration was analyzed by using a UV-vis spectrophotometer (Agilent 8453) at the maximum absorbance wavelength dye. The degradation efficiency and capacity of dye were calculated by the following equations:

(1) $$\begin{array}{c} \text{Degradation efficiency}\left(\text{\%}\right)=\frac{C_{o}-C_{t}}{C_{o}}\times 100\%, \end{array}$$

(2) $$\begin{array}{c} \text{Degradation capacity}\left(\text{mg}/\text{g}\right)=\frac{\left(C_{o}-C_{t}\right)\times V}{m}. \end{array}$$

The degradation rate of dye was determined by fitting the degradation profile with the following first-order kinetics model:

(3) $$\begin{array}{c} \text{ln}\frac{C_{o}}{C_{t}}=k_{ap}\times t, \end{array}$$

where *k*_*ap*_ (s^−1^) is the rate constant, *C*_*o*_ is the initial concentration of dye and *C*_*t*_ is the concentration of dye in time, *V* is the volume of dye solution (L), *m* is the mass of the adsorbent (g), and *t* is the reaction time (min).

## 3. Results and Discussion

### 3.1. Physiochemical Characterization

**Figure 1:**
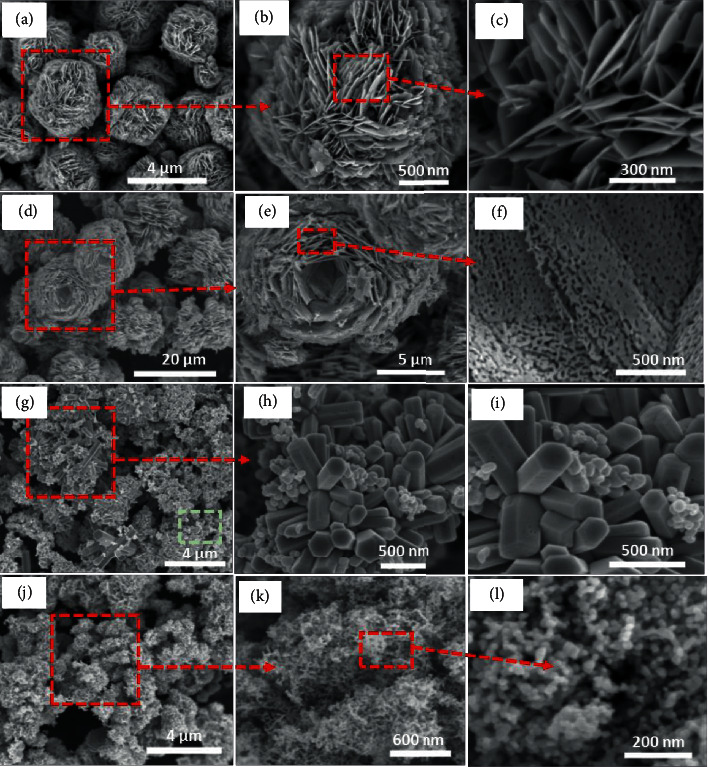
SEM images of the as-synthesized samples at the different scale bars: (a–c) CF-ZnO; (d–f) RF-ZnO; (g–i) R-ZnO; and (k–l) N-ZnO.

Morphology, structure, and particle size are very crucial to the photocatalytic reaction of ZnO because they are related to the surface area and pore volume, which will give an advantage for the adsorption, diffusion, and reaction of organic compounds on the surface of ZnO [25]. The SEM images of as-prepared ZnO samples are shown in Figure 1. CF-ZnO had a hierarchical structure like a carnation flower with a size of about 3-4 *μ*m, and it was assembled from many thin uniform petals with a thickness of about 10–20 nm (Figures 1(a)–1(c)). Meanwhile, the morphology of RF-ZnO was like a beautiful rose, and the microstructures were about 15–20 *μ*m in size. They were composed of many petals with a thickness of about 10–15 nm, composed of many holes of 30–40 nm in size (Figures 1(d)–1(f)). These results imply that the orientation of trisodium citrate can produce the hierarchical structure of ZnO by the simple precipitation method without a hydrothermal process, while the hydrothermal process together with the presence of urea can result in the formation of holes for the hierarchical structure.

Unlike CF-ZnO and RF-ZnO, the R-ZnO microstructure had an indeterminate shape, and it was composed of many hexagonal rods and a few spherical particles (Figures 1(g)–1(i)). Since the morphology of ZnO depended on the synthesis methods [34–37], the reaction agent [38], reaction temperature, aging, and hydrothermal time, etc., for ZnO nanorods, the morphology was strongly influenced by aging time and stirring rate [39]. The aging time was related to the growth of crystallite and aggregation of crystallites, and the prolongation of aging time could increase the perfection and uniformity of particles, while the stirring rate was related to the saturation of the substrates and nucleation in the solution. The slow stirring rate led the slow reaction rate, resulting in a decrease in crystal nuclei and an increase in the crystal size. However, the fast stirring rate could reduce the local saturation, leading to an increase in the crystal nuclei and a decrease in crystal size. In this study, a few spherical particles in the R-ZnO sample could be attributed to rapid stirring rate and short aging time, whereas the N-ZnO microstructure was observed as clouds with 2-3 *μ*m size, and it was made up of ZnO nanoparticles with approximately 20–30 nm (Figures 1(k)–1(m)). These results imply that if HMTA is used as the precipitation agent instead of NaOH for synthesis of CF-ZnO under fast reaction, the mixed structure of rod and nano can be produced, while the addition of trisodium citrate leads to suppress the formation of a rod shape, which promotes the generation of small particles.

**Figure 2:**
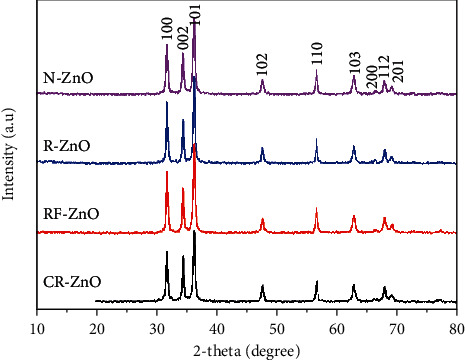
XRD patterns of the CF-ZnO, RF-ZnO, R-ZnO, and N-ZnO samples.

The XRD results of as-prepared ZnO samples are presented in Figure 2. ZnO crystallites in all samples had the same hexagonal wurtzite-type structure with lattice parameters in accordance with the standardized JCPDS 19-1458 card [12]. These diffraction peaks corresponding to (100), (002), (101), (102), (110), (103), (200), (112), and (201) planes were observed for pure Zn, but RF-ZnO and R-ZnO showed a higher intensity than N-ZnO and CR-ZnO. In addition, there were no characteristic peaks for impurity phases such as Zn, Zn(OH)_2_, and Zn(OH)_2_CO_3_, indicating the high purity of the as-prepared ZnO samples.

The average crystallite size (*D*) of ZnO samples was calculated from X-ray line broadening of the diffraction peaks by using Debye–Scherrer's equation [40].

(4) $$\begin{array}{c} D=\frac{K\lambda }{\beta \;\cos\;\theta }, \end{array}$$

where *K* is a dimension shape factor (the typical value of approximately 0.9); *λ* is the wavelength of X-ray used (0.15405 nm), *β* is the broadening of the diffraction line measured in radians at half of its maximum intensity (FWHM), and *θ* is Bragg's diffraction angle [28]. Based on the calculation, the average crystallite sizes of ZnO samples were 17.3, 17.5, 22.5, and 17.8 nm for CF-ZnO, RF-ZnO, R-ZnO, and N-ZnO, respectively (Table 2). The inconsistency in crystallite sizes from XRD and SEM results might be assigned to the hierarchical structure of CF-ZnO and RF-ZnO samples, and their 2D-structure of petals, 1D-structure of R-ZnO, and 3D-structure of N-ZnO otherwise. These results imply that the crystallinity and crystallite size of ZnO are dependent on the synthesis process.

**Figure 3:**
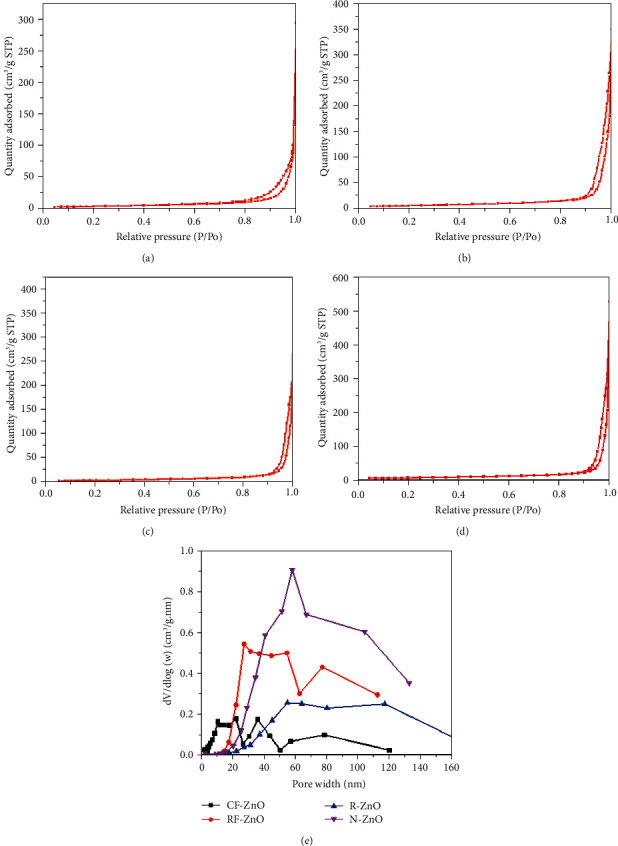
N_2_ adsorption/desorption isotherms of (a) CF-ZnO, (b) RF-ZnO, (c) R-ZnO, and (d) N-ZnO, respectively, and (e) pore size distributions.

Figure 3 shows the typical N_2_ adsorption/desorption isotherms and the corresponding pore size distribution curves of as-synthesized ZnO samples. According to the IUPAC classification, the isotherms of all ZnO samples were defined as the combination of type III and IV with a small hysteresis loop (H_3_), in Figures 3(a)–3(d), which were characteristic to low mesoporous materials [41]. The specific surface area of RF-ZnO was larger than that of CF-ZnO, and the specific surface area of N-ZnO was larger than that of R-ZnO. As seen in Table 2, the BET surface areas of CF-ZnO, RF-ZnO, R-ZnO, and N-ZnO were 16.1, 24.4, 18.1, and 26.4 m^2^/g, respectively. The N-ZnO sample had a much higher surface area than CF-ZnO and R-ZnO due to small particle size and large pore volume rather than large pore with of N-ZnO.

The hysteresis loops of four ZnO samples were observed at high relative pressure (*p*/*p*_*o*_) of 0.81–1.0, suggesting the presence of large pores. In this study, the pore size distribution of ZnO samples calculated by desorption branch of the N_2_ adsorption/desorption isotherm by the Barrett–Joiner–Halenda (BJH) method, and the results are shown in Figure 3(e). The pore size distributions of ZnO samples were relatively wide. The pore size distribution of CF-ZnO was in three regions, 7–22, 25–45, and 60–120 nm. Meanwhile, RF-ZnO has 2 regions, 20–55 and 65–90 nm. Unlike the abovementioned materials, only one peak was observed for each R-ZnO and N-ZnO samples, concentrating in 40–140 and 30–120 nm, respectively, but the peak of N-ZnO was higher than that of R-ZnO. It is presumed that the large pore size is favorable for penetration of organic molecules into the structure of ZnO, but it can prevent the light from entering deep into the solution.

**Figure 4:**
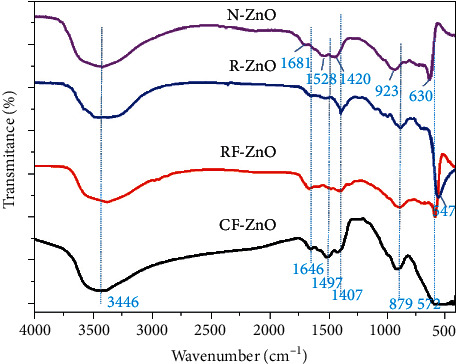
FT-IR spectra of the as-prepared ZnO samples.

The characteristic functional groups and the formation of ZnO were examined via FT-IR spectra, which were obtained in the range of 400 to 4000 cm^−1^. The FT-IR spectra of ZnO samples prepared by different methods are shown in Figure 4. All the samples showing broad absorption bands at ∼3446 cm^−1^ can be assigned to the stretching vibration of the intermolecular hydrogen bond (O-H) existing between the absorbed water molecules (H-O-H), which indicates the higher amount of the hydroxyl group [41]. The bands at ∼1681 and 1646 cm^−1^ can be associated with the bending vibrations of H_2_O molecules [42]. The absorption bands at ∼1407, ∼930, and ∼879 cm^−1^ might due to the vibration of the C=O bond in CO_2_ adsorbed on the surface of ZnO [43]. The Zn-O bond could exhibit the adsorption peaks in the range of 650–400 cm^−1^ [44]. The characteristic peaks at 630, 572, and 547 cm^−1^ become stronger, indicating the formation of Zn-O stretching vibration of N-ZnO, RF-ZnO, and R-ZnO samples, respectively. The shift and change of intensity of peaks could be assigned to the change in the lattice parameter, and due to the change in crystallite size of ZnO samples otherwise [11]. However, for the CF-ZnO sample, the peaks at about 650–500 cm^−1^ are relatively weak and not shown in the FT-IR spectrum. It could be attributed to the formation of very thin petals, resulting in weaker vibration intensity of the Zn-O bonds in the CF-ZnO sample.

**Figure 5:**
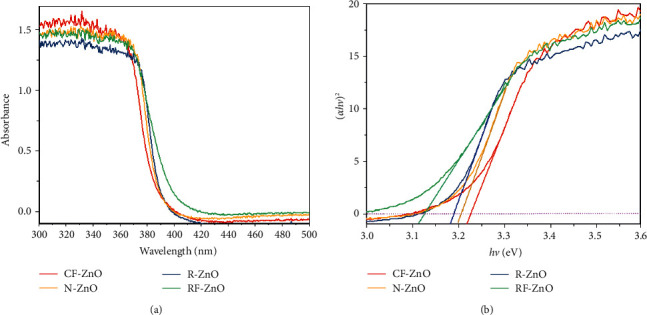
UV-Vis diffuse reflectance spectra and Tauc's plots of the as-prepared samples.

Figure 5 shows the DR/UV-vis spectra and Tauc's plots of the as-prepared ZnO samples. All the ZnO samples showed a relatively high absorption at wavelengths from 300–370 nm; then, they significantly decreased at wavelengths from 370–420 nm and showed a stability at above 420 nm, as seen in Figure 5(a). The bandgap energies of the as-prepared samples were estimated by following Tauc's equation [45]:

(5) $$\begin{array}{c} Ahv=A{\left(hv-Eg\right)}^{n}, \end{array}$$

where *α* is the absorption coefficient, *hν* is the photon energy, *A* is a constant, *E*_*g*_ is the bandgap energy. Since ZnO has a direct band structure, the value of *n* is 1/2 in this case, and equation (5) becomes

(6) $$\begin{array}{c} {\left(\alpha hv\right)}^{2}=A\left(hv-E_{g}\right). \end{array}$$

The optical bandgap can be found out by extrapolation of the linear portion in the plot of (*αhν*)^2^ against *hν*, and the results are presented in Figure 5(b). The bandgap energies of CF-ZnO, N-ZnO, R-ZnO, and RF-ZnO were 3.226, 3.207, 3.190, and 3.127 eV, respectively. The bandgap energy of a semiconductor depends on not only the lattice parameters but also the crystallinity, crystallite size, and morphology [46]. The bandgap energy of R-ZnO was larger than that of N-ZnO and CF-ZnO due to the higher crystallinity and larger crystallite size of R-ZnO than others, as seen in Figure 2. Although the crystallite size of RF-ZnO was smaller than that of R-ZnO and N-ZnO, it showed the lowest bandgap energy among as-prepared samples. This could be assigned to the hierarchical structure of CF-ZnO and consists of hole in its petals. However, all samples showed the small bandgap energy, and it can facilitate the absorption of energy from the light of electron to move from the valent band to the conduction band, resulting in the formation of the free radicals (^•^O_2_^−^ and ^•^OH), promoting increase in degradation of organic compounds.

### 3.2. Photocatalytic Study

**Figure 6:**
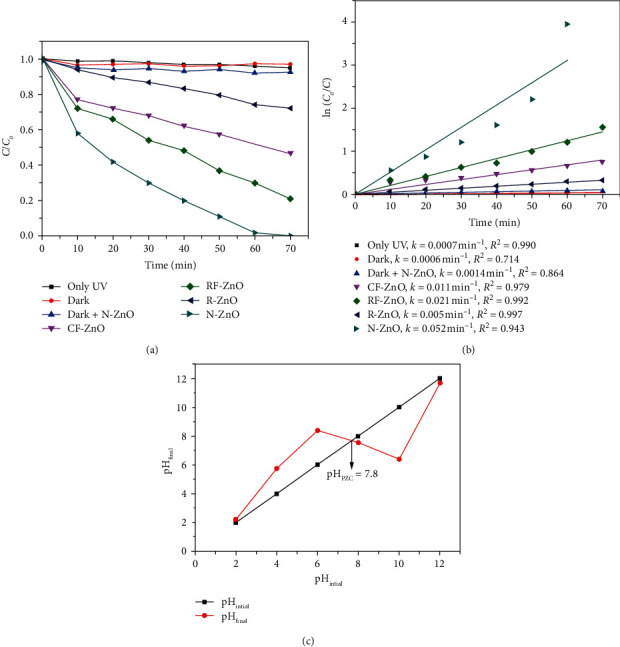
Photodegradation of TA in as-prepared samples, (b) the kinetic curves, and (c) the pH_PZC_ determination of N-ZnO. The reaction conditions: dosage catalyst 0.5 g/L, tartrazine concentration of 20 mg/L, and pH = 6.0.

**Figure 7:**
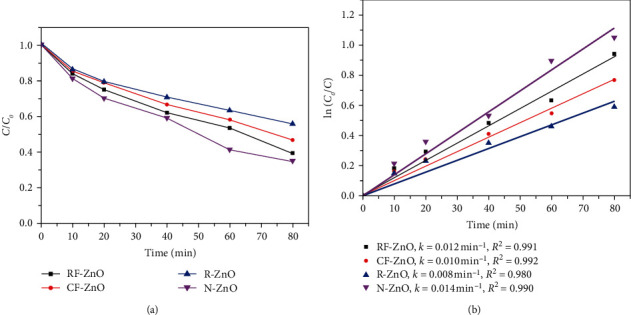
Photodegradation of caffeine in as-prepared samples and (b) the kinetic curves. The reaction conditions: dosage catalyst 0.5 g/L, CAF concentration of 20 mg/L, and pH = 6.0.

The photocatalytic activity of the as-prepared ZnO samples was evaluated by degrading some of the organic pollutants. For this purpose, tartrazine and caffeine were used as model organic pollutants of dyes and medicine, respectively. The reaction conditions were fixed, catalyst dosage was 0.5 g/L, organic compound concentration was 20 mg/L, and solution pH = 6.0, and the results are presented in Figures 6 and 7.

It can be observed that tartrazine is a stable pollutant because it did not degrade in the dark and in the UV irradiation. Also, in the presence of N-ZnO but without UV light, the TA removal was also very low (Figure 6(a)). Although the surface of N-ZnO was positively charged, since solution pH = 6.0 below pH_zpc_ of 7.8 (Figure 6(c)) and tartrazine was an anion dye, the adsorption ability of ZnO was negligible. Tartrazine was virtually not photolytically degraded under UV-light irradiation without the presence of photocatalysts. Nevertheless, in the presence of both excitation energy of UV light and ZnO catalyst, degradation of tartrazine occurred rapidly. The degradation process depends strongly on the type of ZnO. The catalytic ability of R-ZnO was the lowest among as-prepared samples, showing the decomposition efficiency, degradation capacity, and reaction rate of 25%, 8 mg/g, and 0.005 min^−1^, respectively (Figures 6(a) and 6(b)). Although the surface area and the bandgap energy are the parameters that can affect the catalytic activity of photocatalyst, these did not contribute to the enhanced catalytic performances of CF-ZnO and RF-ZnO. These samples showed a higher reaction rate and degradation efficiency than that of R-ZnO, and it might be due to the smaller crystallite size of CF-ZnO and RF-ZnO as compared to R-ZnO (Table 2), and the hierarchical structure of CF-ZnO and RF-ZnO otherwise. As the results, the degradation efficiencies were 47 and 76%, the degradation capacities were 18.8 and 30.4 mg/g, and the reaction rates were 0.011 and 0.021 min^−1^ for CF-ZnO and RF-ZnO, respectively.

The N-ZnO sample exhibited the highest catalytic performance of TA among as-prepared samples. The degradation efficiency reached 100% in 70 min, showing the degradation capacity and reaction rate of 40 mg/g and 0.052 min^−1^, respectively. By considering the bandgap energy and textural properties of as-prepared samples in Table 2, the strong degradation of TA on N-ZnO could be assigned to its small particle size rather than its large surface area, pore volume, and pore with.

Figure 7 shows the photocatalytic degradation of CAF on ZnO samples. Like the result of TA, the N-ZnO sample exhibited the highest performance, while R-ZnO exhibited the lowest performance among as-prepared samples. The degradation efficiency, degradation capacity, and reaction rate of N-ZnO were 65%, 26 mg/g, and 0.014 min^−1^, respectively. These were 40%, 16 mg/g, and 0.008 min^−1^ for R-ZnO, respectively, while the degradation efficiencies and reaction rates were 60% and 0.012 min^−1^ and 50 % and 0.010 min^−1^ for RF-ZnO and CF-ZnO, respectively.

In the previous studies [11, 12], the effect of solution pH on the catalytic performance of ZnO and Ag/ZnO samples was investigated. It was clearly shown that the alkaline medium could enhance the photocatalytic reactions of caffeine and BPA on ZnO and Ag/ZnO catalysts. However, if the solution pH was greater than 11.0, the ZnO material could be dissolved, leading to reduction in the reuse of the catalyst. Therefore, the pH range suitable for photocatalysis was very wide from 6.0 to 11.0. It is relatively suitable for practical application since the pH range of industrial, agricultural, and domestic wastewaters commonly belongs to the alkaline medium.

### 3.3. Degradation of the Different Organic Dyes

**Figure 8:**
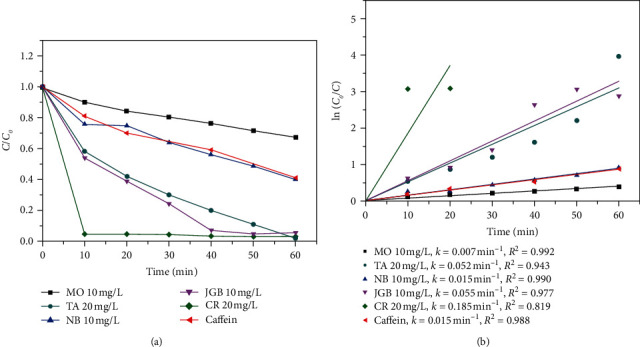
(a) Photodegradation of the different dyes on N-ZnO and (b) the kinetic curves. The reaction conditions: dosage catalyst 0.5 g/L, dye concentration of 20 mg/L, and pH = 6.0.

Since the real wastewaters from textile, rubber, food, and medicine factories commonly contain various types of organic pollutants, to demonstrate the value of as-synthesized ZnO in practical applications, the N-ZnO sample was tested with other organic compounds. The photocatalytic activity of ZnO depends not only on physical properties (morphology, particle size, surface area, pore size, and bandgap energy) but also properties of dyes. Besides TA and CAF, other organic compounds such as methylene orange (MO), Nile blue (NB), Janus green B (JGB), and Congo red (CR) were tested on N-ZnO since it was the most effective catalyst. The reaction conditions were fixed, the catalyst dosage was 0.5 g/L, dye concentration was 10 mg/L, and pH of the solution was 6.0. The results are presented in Figure 8. The N-ZnO sample was shown to be an effective catalyst for the degradation of organic compounds. CR was almost completely degraded in 10 min, and JGB almost completely degraded in 40 min. The degradation efficiencies and reaction rates of CR and JGB were 96.8% and 0.185 min^−1^ and 95.8% and 0.055 min^−1^, respectively, while the reactions of TA, NB, and CAF on N-ZnO were low. Also, MO exhibited the lowest reaction, showing the degradation efficiency and reaction rate of 24% and 0.007 min^−1^, respectively.

## 4. Conclusions

ZnO samples with the different morphologies were successfully prepared by varying synthesis methods and adding a capping agent. All samples had a microstructure and a crystallite of hexagonal wurtzite type. CF-ZnO and RF had a hierarchical structure like a carnation flower and a beautiful rose, respectively. The orientation of trisodium citrate can produce the hierarchical structure of ZnO by the simple precipitation method without the hydrothermal process. However, the hydrothermal process together with the presence of urea can result in the formation of holes in the hierarchical structure. The R-ZnO microstructure was composed of many hexagonal particles and a few spherical particles, whereas the N-ZnO microstructure was observed as clouds with 2-3 *μ*m size, and they were made up of ZnO nanoparticles with approximately 20–30 nm. In the synthesis process of CF-ZnO, if HMTA is used instead of NaOH, the mixed structure of rod and nano can be produced. The addition of trisodium citrate leads to suppression in the formation of the rod shape, which promotes the generation of small particles. The surface area, pore volume, and pore size of N-ZnO were larger, but its particle size was smaller than that of other ZnO samples.

The crystallite size and hierarchical structure contribute to enhanced photocatalytic reaction. The strong degradation of TA on N-ZnO could be assigned to its small particle size rather than its large surface area, pore volume, and pore width, while the large crystallite size may lead to decrease in the activity of R-ZnO. As a result, the degradation performance of TA and CAF was in the order as follows: R-ZnO < CF-ZnO < RF-ZnO < N-ZnO. The degradation efficiency, degradation capacity, and reaction rate for TA and CAF on N-ZnO were 100%, 40 mg/g, and 0.052 min^−1^ and 65%, 26 mg/g, and 0.014 min^−1^, respectively. N-ZnO appears to be a potential photocatalyst with good degradation efficiency for organic compounds (TA, CAF, MO, NB, JGB, and CR).

## Figures and Tables

**Table 1:** The basic characteristics of tartrazine and caffeine.

Chemical	Tartrazine	Caffeine
Chemical formula	C_16_H_9_N_4_Na_3_O_9_S_2_	C_8_H_10_N_4_O_2_
Chemical class	Azo dye	Methylxanthine alkaloid
Molecular weight (g/mol)	534.4	194.19
Color	Yellow	Colorless
*λ*_max_ (nm)	428	273
C.I. number	19140	—
Nature	Anion dye	—
Molecular structure	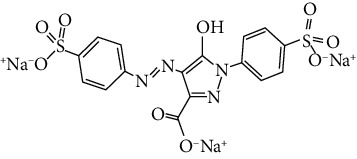	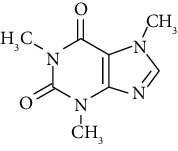

**Table 2:** Textural properties of as-prepared ZnO samples.

Sample	*E*_g_ (eV)	Average crystallite size *D* (nm)	*S*_BET_ (m^2^/g)	BJH pore volume (cm^3^/g)	Average pore width (nm)
CF-ZnO	3.226	17.3	16.1	0.155	26.9
RF-ZnO	3.127	17.5	24.4	0.408	47.9
R-ZnO	3.190	22.5	18.1	0.314	53.1
N-ZnO	3.207	17.8	26.4	0.481	53.7

## Data Availability

The research data used to support the findings of this study are included within the article.
